# Association of RAB5 overexpression in pancreatic cancer with cancer progression and poor prognosis via E-cadherin suppression

**DOI:** 10.18632/oncotarget.14703

**Published:** 2017-01-17

**Authors:** Takamichi Igarashi, Kenichiro Araki, Takehiko Yokobori, Bolag Altan, Takahiro Yamanaka, Norihiro Ishii, Mariko Tsukagoshi, Akira Watanabe, Norio Kubo, Tadashi Handa, Yasuo Hosouchi, Masahiko Nishiyama, Tetsunari Oyama, Ken Shirabe, Hiroyuki Kuwano

**Affiliations:** ^1^ Department of Hepatobiliary and Pancreatic Surgery, Gunma University Graduate School of Medicine, Maebashi, Gunma, Japan; ^2^ Integrative Center of General Surgery, Gunma University Hospital, Maebashi, Gunma, Japan; ^3^ Division of Integrated Oncology Research, Gunma University Initiative for Advanced Research, Maebashi, Gunma, Japan; ^4^ Department of Diagnostic Pathology, Gunma University Graduate School of Medicine, Maebashi, Gunma, Japan; ^5^ Department of Surgery and Laparoscopic Surgery, Gunma Prefecture Saiseikai-Maebashi Hospital, Maebashi, Gunma, Japan; ^6^ Department of Molecular Pharmacology and Oncology, Gunma University Graduate School of Medicine, Maebashi, Gunma, Japan

**Keywords:** RAB5, pancreatic cancer, epithelial mesenchymal transition, E-cadherin, cancer progression

## Abstract

Pancreatic cancer is a common type of cancer with poor prognosis worldwide. Postoperative survival depends on the existence of metastasis. Elucidation of the mechanism underlying cancer progression is important to improve prognosis. The RAS-associated protein RAB5 activates intracellular membrane trafficking, and RAB5 expression is correlated to progression and epithelial mesenchymal transition in various cancers.

The expression of RAB5 and E-cadherin in 111 pancreatic cancer samples was investigated by immunohistochemical staining, and the relationship among RAB5 expression, clinicopathological factors, and E-cadherin expression was assessed. Furthermore, RAB5 suppression analysis by siRNA was performed to determine the roles of RAB5 in morphological change, proliferation potency, cell migration ability, and invasiveness of the pancreatic cancer cell line.

High RAB5 expression correlated with the presence of lymphatic invasion and venous invasion and low E-cadherin expression. Patients with high RAB5 expression had a poorer prognosis than those with low RAB5 expression. RAB5 suppression in pancreatic cancer cells enhanced E-cadherin expression; changed cell morphology from spindle to round; and inhibited proliferation, invasion, and cell migration.

RAB5 contributes to poor prognosis and progression in pancreatic cancer patients. It may be a promising candidate for individualized therapy in refractory pancreatic cancer.

## INTRODUCTION

Pancreatic cancer is a common type of cancer with poor prognosis worldwide [1]. The 5-year survival rate for pancreatic cancer is approximately 10% [2]. Surgical therapy is the only effective curative treatment for pancreatic cancer, and postoperative survival has been shown to be dependent on the existence of invasion and metastasis [3] [4]. Therefore, it is important to elucidate the mechanism underlying cancer progression in pancreatic cancer to improve patient prognoses.

Epithelial to mesenchymal transition (EMT) activates embryonic development and cancer progression [5]. EMT is a complex molecular and cellular program by which epithelial cells shed their differentiated characteristics, i.e., cell adhesion and cellular polarity, and acquire mesenchymal features, including motility, invasiveness, and a heightened resistance to apoptosis. These phenotypes contribute to tumor progression and intra-tumoral heterogeneity [6]. The induction of EMT in immortalized human mammary epithelial cells results in the gain of stem cell properties [7]. In breast cancer, there are reports of an emerging relationship between EMT and cancer stem cells [8]. Zheng et al. reported the importance of combining EMT inhibition with chemotherapy for the treatment of pancreatic cancer [9].

Kirsten rat sarcoma viral oncogene homolog (*KRAS*) is a member of the *RAS* gene family. Among the few pancreatic cancer-related genetic mutations, *KRAS* is very common [10]. The mutation of *KRAS* in pancreatic cancer induces long-term activation of the P21 RAS protein, which is a small guanosine triphosphatase (GTPase). This activation enlivens many cellular processes such as proliferation, invasion, transformation, and survival [11]. A meta-analysis of pancreatic cancer patients showed a significant association between *KRAS* gene mutations and overall survival [12]. The small GTPase RAB5 is an RAS-associated protein that is known to function as the master regulator of endocytosis. Luo et al. reported that RAB3D activates the Akt pathway and induces the EMT process in colorectal cancer cells [13]. RAB5 also stimulates EMT induction via the endocytosis of transforming growth factor-beta (TGF-β) receptor [14]. RAB5 promotes cell invasion and migration by stimulating focal adhesion turnover [15]. RAB5 is highly expressed in various types of human malignancies, and its expression correlates with tumor progression and poor prognosis in many cancers, including breast cancer and ovarian cancer [16]. These studies indicate that RAB5 is a fundamental cancer-associated gene and a potential factor for diagnosis and treatment. In pancreatic cancer, patients with a high RAB27B expression have significantly poorer prognosis, and a significant correlation between RAB27B and p53 expression has been observed [17]. However, there is no report of the clinical significance of RAB5 expression in pancreatic cancer.

We aimed to clarify the function of RAB5 as an EMT regulators in pancreatic cancer cell lines *in vitro* and to determine the clinical significance of RAB5 and E-cadherin in pancreatic cancer. Therefore, we performed an immunohistochemical analysis to evaluate the relationships among RAB5, E-cadherin, and clinicopathological factors in clinical pancreatic cancer samples. We also examined the *in vitro* effects of small-interfering RNA (siRNA)-correlated RAB5 suppression on E-cadherin expression, morphology, proliferation, invasion, and migration of human pancreatic cancer cells.

## RESULTS

### Immunohistochemical staining of RAB5 and E-cadherin in pancreatic cancer tissues

**Figure 1:**
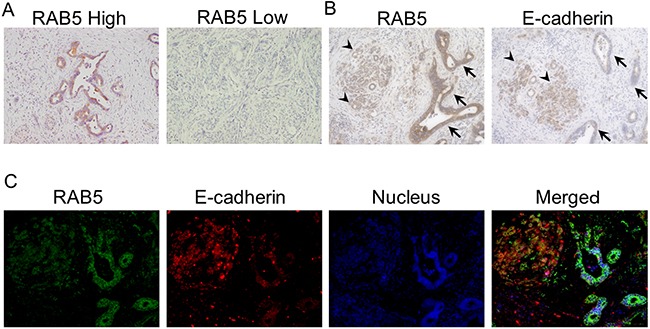
Immunohistochemical staining of RAB5 and E-cadherin in primary pancreatic cancer samples **A**. Examples of high and low RAB5 expression in primary pancreatic cancer specimens (200×). **B**. An example of high RAB5 expression and low E-cadherin expression in a primary pancreatic cancer specimen (400×). Arrowhead, normal acinar cells; arrow, atypical ducts. **C**. Fluorescence immunohistochemical analysis of RAB5 and E-cadherin expression in a representative pancreatic cancer tissue (400×).

RAB5 expression was evaluated by immunohistochemistry in 111 pancreatic cancer samples. The staining was mainly observed at the cytoplasm in positive cases. Fifty (45%) pancreatic cancer specimens were assigned to the low RAB5 expression group and 61 (55%) were assigned to the high RAB5 expression group; 63 (57%) pancreatic cancer specimens were assigned to the high E-cadherin expression group and 48 (43%) were assigned to the low E-cadherin expression group (Figure 1A). High RAB5 expression and low E-cadherin expression of pancreatic cancer cells are shown in the serial section (Figure 1B). The expression of the epithelial marker E-cadherin was examined to validate the relationship between EMT and RAB5 in a representative identical pancreatic cancer section. The results indicate that RAB5 expression inversely correlated with E-cadherin levels in only pancreatic cancer parts (Figure 1C).

### Association between RAB5 expression and clinicopathological features of pancreatic cancer

The correlations between RAB5 expression and patients’ clinicopathological characteristics (age, gender, histology type, tumor size, tumor stage, lymph node metastasis, lymphatic invasion, venous invasion, peri-neural invasion, infiltration, pathological stage, and recurrence) and E-cadherin levels are shown in Table 1. The results indicate that patients with high RAB5 expression in tumors showed significant lymphatic (*P* = 0.012) and venous (*P* = 0.019) invasions, as well as low E-cadherin expression (*P* = 0.010). However, no significant differences were observed in age, gender, histology type, tumor size, tumor stage, lymph node metastasis, peri-neural invasion, infiltration, pathological stage, and recurrence.

**Table 1:** Clinicopathological characteristics and E-cadherin expression in pancreatic cancer patients stratified by RAB5 expression

Factors	RAB5expression		
	Low (n=50)	High (n=61)	p-value
Age			0.902
<70	26	31	
≧70	24	30	
Gender			0.812
male	29	34	
female	21	27	
Histology type			0.107
well	9	6	
mod,por	30	50	
Tumor size stage			0.088
TS1,2	34	50	
TS3,4	16	11	
Tumor stage			0.505
T1-3	31	34	
T4	19	27	
Lymph node metastasis			0.135
N0	17	13	
N1	33	48	
Lymphatic invasion			0.012*
ly0	5	0	
ly1,2,3	45	60	
Venous invasion			0.019*
v0	8	2	
v1,2,3	42	58	
Peri-neural invasion			0.060
ne0,1	19	13	
ne2,3	31	47	
Infiltration			0.068
α	2	0	
β	37	37	
γ	11	23	
pStage(UICC)			0.088
I,IIA	17	12	
IIB,III,IV	33	49	
Recurrence			0.805
absent	35	44	
present	15	17	
E-cadherin expression			0.010*
high	35	28	
low	15	33	

*p<0.05.

well: well differentiated, mod: moderately differentiated, por: poorly differentiated.

### Prognostic significance of RAB5 expression in pancreatic cancer

**Figure 2:**
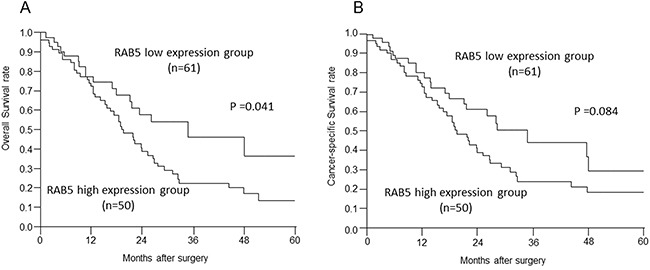
Relationships between postoperative survival and RAB5 and E-cadherin expression **A, B**. Overall survival and cancer-specific survival of pancreatic cancer patients according to RAB5 expression (*P* = 0.041, *P* = 0.084).

The high RAB5 expression group had significantly poorer prognosis than the low RAB5 expression group with respect to overall survival (*P* = 0.041, Figure 2A). Cancer-specific survival had tendency for poorer prognosis (*P* = 0.084, Figure 2B). There was no significant difference in the relapse-free survival (*P* = 0.740).

For overall survival, RAB5 expression was a prognostic factor for poor survival in univariate analysis. Interestingly, other existing clinicopathological factors were not significantly associated with poor prognosis, whereas the detection of high RAB5 expression in pancreatic cancer tissues remained prognostically significant with regard to overall survival (Table 2; RR = 1.32, 95% CI = 1.02–1.74, *P* = 0.0376).

**Table 2:** Univariate analyses of patients’ clinicopathological characteristics affecting overall survival rate after surgery

Factors	Univariate analysis		
	RR	95%CI	p-value
Age			
(<70 vs. ≧70)	1.00	0.78-1.28	0.999
Gender			
(male vs. female)	0.78	0.47-1.29	0.345
Tumor size stage			
(TS1,2 vs. TS3,4)	0.830	0.63-1.12	0.218
Tumor stage			
(T1-3 vs. T4)	1.51	0.90-2.48	0.112
Lymph node metastasis			
(absent vs. present)	1.01	0.58-1.88	0.961
Lymphatic invasion			
(absent vs. present)	1.13	0.35-6.89	0.866
Venous invasion			
(absent vs. present)	1.33	0.55-4.40	0.564
Peri-neural invasion			
(ne0,1 vs. ne2,3)	1.24	0.72-2.26	0.446
pStage(UICC)			
(I, IIA vs. IIB, III, IV)	0.998	0.57-1.85	0.995
RAB5 expression			
(Low vs. High)	1.32	1.02-1.74	0.0376*

RR;Relative risk, CI;Confidence interval, * p<0.05.

### Expression of RAB5 in pancreatic cancer cell lines and depletion of RAB5 using siRNA

**Figure 3:**
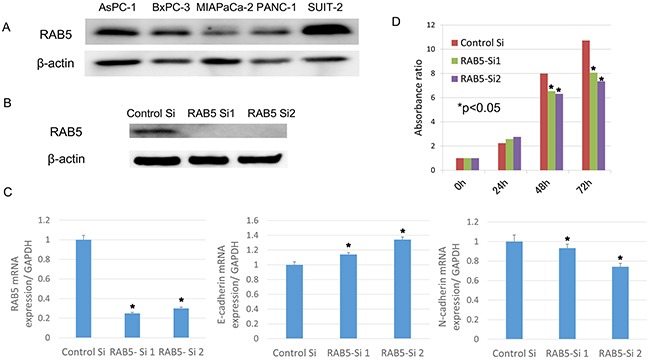
Functional analysis of RAB5 by small-interfering RNA (siRNA) **A**. RAB5 protein levels were measured by western blotting for pancreatic cancer cell lines. **B**. RAB5 expression in RAB5 siRNA-transfected and untreated SUIT-2 cells assessed by western blotting. **C**. RAB5, E-cadherin, and N-cadherin mRNA expression in RAB5 siRNA-transfected and untreated SUIT-2 cells assessed by RT–qPCR (*P < 0.05). **D**. Proliferation assay in RAB5 siRNA-transfected and untreated SUIT-2 cells assessed by the Cell Counting Kit-8 assay (*P < 0.05). Si: siRNA.

RAB5 protein expression was observed in all pancreatic cancer cell lines [i.e., AsPC-1, BxPC-3, MIAPaCa-2, PANC-1, and SUIT-2 (Figure 3A)]. We used siRNA to knock down RAB5 expression in the pancreatic cancer cell line SUIT-2, which showed the highest expression of RAB5, to determine the contribution of RAB5 to proliferation, invasion, and migration. The suppression of RAB5 by siRNA1 and siRNA2 was demonstrated by western blotting and quantitative real-time reverse transcription–polymerase chain reaction (RT–qPCR) (Figure 3B, 3C).

### RAB5-specific siRNA inhibits cancer cell proliferation *in vitro*

We examined the relevance of RAB5 to the expression of the EMT-correlated marker E-cadherin. The suppression of RAB5 significantly increased E-cadherin expression and decreased N-cadherin expression in SUIT-2 cells (Figure 3C). These cells transfected with RAB5-specific siRNA showed significantly reduced proliferation compared with control siRNA-transfected cells (Figure 3D).

### RAB5 activated cell morphology, invasion, and migration in SUIT-2 cells

**Figure 4:**
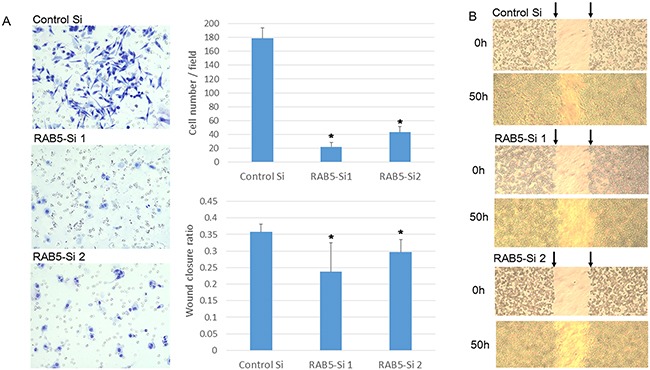
Invasion and migration assays of SUIT-2 cells treated by RAB5 siRNA **A**. Invasion assay of RAB5 siRNA-transfected and untreated SUIT-2 cells (*P < 0.05). **B**. Wound healing assay of RAB5 siRNA-transfected and untreated SUIT-2 cells (*P < 0.05). Si: siRNA.

We assessed the role of RAB5 in cell morphology, invasion, and migration. RAB5 knockdown significantly changed the cell morphology from spindle to round and reduced cell invasiveness compared with that of control siRNA-transfected cells (Figure 4A). As revealed in the wound-healing assay, RAB5 knockdown suppressed cell migration in comparison with that of control cells (Figure 4B).

## DISCUSSION

In this study, we showed that high RAB5 expression was associated with cancer progression and poor prognosis of primary pancreatic cancer samples. Moreover, high RAB5 expression was correlated to low E-cadherin expression. In RAB5 suppression analysis *in vitro*, the cell morphology was changed from spindle to round. In addition, compared with control cells, the ability of proliferative potency, invasiveness, and migration was reduced in RAB5-suppressed cells via the induction of E-cadherin expression.

Previous studies have demonstrated that oncogenic GTPase *KRAS* mutation is involved in the initiation of pancreatic intraepithelial neoplasia and activates the progression of pancreatic cancer, especially pancreatic ductal adenocarcinoma [10] [11] [12] [18]. The small GTPase RAB5 is a RAS-associated protein that is an important regulator of early endocytosis and is known to influence cell migration and tumor dissemination, possibly through the regulation of β1-integrin traffic [19]. RAB5 has three isoforms (RAB5A, B, and C) [20, 21], and its expression is elevated in non-small-cell lung cancer, hepatocellular carcinoma, and ovarian cancer. RAB5B has been shown to be activated in malignant melanoma, while RAB5C enhances epidermal growth factor-induced progression of breast cancer [21, 22]. The present study indicates that RAB5 may represent a novel therapeutic factor and tumor marker. The detection of RAB5 surpassed the existing clinicopathological factors in identifying pancreatic cancer patients at high risk of poor prognosis.

Recently, intracellular trafficking stimulated by the RAB family has been suggested to correlate with epithelial carcinogenesis by controlling cell polarity, cell morphology, proliferation, cell motility, and invasion [23]. RAB25 and RAB27 have been reported to be expressed in cancer cells and to promote cancer progression in pancreatic cancer [24]. In this study, we clarified that the suppression of RAB5 in pancreatic cancer cells changed morphology and reduced the ability of proliferation, invasion, and migration. Consistent with these observations, it has been reported that RAB5 is required to promote migration, invasion, and metastasis in several cancers [16]. Moreover, microRNA-101-altered RAB5 suppression in hepatocellular carcinoma is associated with apoptosis induction and downregulation of proliferation and migration ability [25]. With respect to developing new molecular cancer therapies, RAB5 may be a promising candidate for individualized therapy in refractory pancreatic cancer.

In pancreatic cancer, EMT contributes to the dissemination of cancer cells and stimulates invasion and metastasis [26] [27]. Moreover, current evidence suggests that EMT of pancreatic cancer cells is involved in chemotherapy resistance [9] [28]. Jin et al. demonstrated that Rab 5C-dependent endocytosis activates resistance to chemotherapy [29]. It is expected that the development of specific factors inhibiting RAB5-correlated EMT will decrease invasiveness and drug resistance and will contribute to longer survival of patients with pancreatic cancer.

In our clinical specimens, the epithelial marker E-cadherin was suppressed in pancreatic cancer cells expressing RAB5; moreover, E-cadherin expression was induced and N-cadherin expression was inhibited in RAB5-suppressed pancreatic cancer cells. E-cadherin is well known to be suppressed in EMT induction via several EMT-correlated signals, including TGF-β signals, hypoxia, Notch, Receptor tyrosine kinases, Wnt signals, integrins, and MMPs [30]. A previous study showed that RAB5 suppressed E-cadherin via RAB5-correlated TGF-β signal transduction [31]. Moreover, McLean et al. pointed that RAB5-positive early endosomes stimulate intracellular trafficking of EMT-inducible TGF-β receptor II and Smad signaling activation [32]. In addition, RAB5 has been reported to be a critical player in hypoxia-driven tumor cell migration, invasion, and metastasis [33]. From these observations, we predicted that the correlation of E-cadherin suppression and RAB5 expression in pancreatic cancer cells may be caused by EMT induction via TGF-β signal activation and hypoxia.

In conclusion, RAB5 contributes to shorter survival time in pancreatic cancer patients. The evaluation of RAB5 and E-cadherin expression in pancreatic cancer may be a useful predictor of cancer progression and poor prognosis. Moreover, high RAB5 expression is associated with low E-cadherin expression in clinical pancreatic cancer samples, and RAB5 suppression activates the morphology and ability of proliferation, invasion, and migration of pancreatic cancer cell lines. Our results suggest that RAB5 is a promising molecular factor for regulating pancreatic cancer progression via EMT induction.

## MATERIALS AND METHODS

### Patients and samples

An immunohistochemical analysis was performed on 111 pancreatic cancer patients who had undergone potentially curative surgery at the Gunma University and Gunma Prefecture Saiseikai-Maebashi Hospital between 1999 and 2012 (Table 1). Patients ranged in age from 36 to 87 years. The tumor stage was classified according to the seventh Tumor Node Metastasis (TNM) classification of the Union for International Cancer Control. The patients did not undergo pre-operative chemotherapy. Ninety patients (81%) were given adjuvant chemotherapy, and were administered gemcitabine, TS-1^®^, or UFT^®^ monotherapy. Seventy-nine patients (71%) had recurrence, and 59 of these 79 patients were administered additional chemotherapy with gemcitabine and TS-1^®^, gemcitabine monotherapy, TS-1^®^ monotherapy, or UFT^®^ monotherapy. All patients signed written informed consent forms as required by our institutional guidelines.

### Immunohistochemical staining

A 2-μm section was cut from each paraffin block of pancreatic cancer tissue. All sections were incubated at 60°C for 60 min and deparaffinized in xylene, rehydrated, and incubated with fresh 0.03% hydrogen peroxide in 100% methanol for 60 min at room temperature to block endogenous peroxidase activity. After rehydration using a graded series of ethanol treatments, the sections were heated in boiled water and soaked in Immunosaver (Nishin EM, Tokyo, Japan) at 98°C for 45 min. Nonspecific binding sites were blocked by incubating with Protein Block Serum-Free (DAKO, Carpinteria, CA, USA) for 60 min. A rabbit monoclonal anti-RAB5 (EPR 5438) antibody (Abcam, Cambridge, UK) and mouse monoclonal anti-human E-cadherin (HECD-1) antibody (TAKARA BIO, Otsu, JAPAN) were applied at a dilution of 1:800 and 1:500, respectively, for 24 h at 4°C. The primary antibody was visualized using the Histofine MAX-PO (Multi) Kit (Nichirei, Tokyo, Japan), according to the instruction manual. The chromogen 3,3′-diaminobenzidine tetrahydrochloride (DAB) was applied as a 0.02% solution containing 0.005% H_2_O_2_ in 50 mM ammonium acetate-citrate acid buffer (pH 6.0). The sections were lightly counterstained with Mayer's hematoxylin and mounted. Negative controls were established by replacing the primary antibody, and no detectable staining was evident.

On immunohistochemical staining, some cases were found to be positive and others negative, both among cancer tissues and normal cells. In cancer tissues, heterogeneity was observed between the tumor center and peri-frontal area, but this was not observed in normal cells.

In the past, at the time of immunohistochemical staining, we did not define unfixed time interval after resection definitely. The immunointensity of RAB5 and E-cadherin in normal pancreatic tissues suppressed with the unfixed time interval after operation (Supplementary Figure 1, 2). Thus, the intensity of RAB5 and E-cadherin in normal pancreatic acinar cells was defined as the internal control for evaluation (score 0). Compared with the intensity of the control acinar cells, the immunointensity of RAB5 and E-cadherin staining was scored as −2, −1, 0, +1, or +2 at the tumor center and peri-frontal area (i.e., invasion front). In some cases, there were score differences between the tumor center and peri-frontal area; therefore, the evaluation scores were defined by subtracting the tumor center score from the peri-frontal area score. The score was used for analyzing the relationship among RAB5, E-cadherin, and clinicopathological factors. All samples were then categorized as score −2, −1, 0, +1, +2, or +3. For RAB5, scores −1 and 0 were considered as the low expression group, while scores +1, +2, and +3 were considered as the high expression group. For E-cadherin, scores −2 and −1 were considered as the low expression group, while scores 0 and +1 were considered as the high expression group (Supplementary Table 1).

### Fluorescent immunohistochemistry

The sections were prepared, and endogenous peroxidase was blocked as described above. The sections were then boiled in citrate buffer (pH 6.4) for 15 min in a microwave. Nonspecific binding sites were blocked by incubation with Protein Block Serum-Free reagent for 30 min, and the sections were incubated with the primary antibodies against RAB5 (1:800) and E-cadherin (TAKARA BIO, Otsu, JAPAN) for 3 h at room temperature. Multiplex covalent labeling (RAB5, Fluorescein; E-cadherin, Cyanine 3) with tyramide signal amplification (Opal™ 3-Plex Kit; PerkinElmer) was performed, according to the manufacturer's protocol. All sections were counterstained with DAPI and examined under an All-in-One BZ-X710 fluorescence microscope (KEYENCE Corporation, Osaka, JAPAN).

### Cell culture

The human pancreatic cancer cell lines AsPC-1, BxPC-3, MIAPaCa-2, PANC-1, and SUIT-2 were used in this study. All cells were obtained from RIKEN BRC through the National Bio-Resource Project of MEXT, Tokyo, Japan. The cells were cultured in RPMI 1640 medium (Wako, Osaka, Japan) supplemented with 10% FBS and 1% penicillin–streptomycin (Invitrogen, Carlsbad, CA, USA) in a humidified 5% CO_2_ incubator at 37°C.

### siRNA transfection

RAB5-specific siRNA was purchased from Gene Design (Ibaraki, Osaka, Japan). SUIT-2 cells were used at a density of 5.0 × 10^5^ cells per well in 100 µl of Opti-MEM I Reduced Serum Medium (Invitrogen, Carlsbad, CA, USA). In total, 20 nM of RAB5-specific siRNA 1 and 2 and scrambled siRNA as a negative control was mixed into cells; siRNA was transfected using an electroporator CUY-21 EDIT II (BEX, Tokyo, Japan), according to the manufacturer's instructions. Poring pulses were applied at 125 V (pulse length, 10.0 ms; 1 pulse interval, 40.0 ms), and transfer pulses were applied at 10 V (pulse length, 50.0 ms; 5 pulses interval, 50.0 ms). After 24–72 h of incubation in a humidified atmosphere (37°C and 5% CO_2_), the experiments were performed.

### Protein extraction and western blot analysis

Western blotting was performed to confirm the expression of RAB5 and β-actin proteins in pancreatic cancer cell lines. Total protein was extracted from AsPC-1, BxPC-3, MIAPaCa-2, PANC-1, and SUIT-2 using the RIPA buffer. The proteins were separated by sodium dodecyl sulfate polyacrylamide gel electrophoresis using 12.5% Bis–Tris gels and transferred to PVDF membranes. The membranes were incubated overnight at 4°C with rabbit monoclonal antibodies against RAB5 (1:1000; Abcam, Cambridge, UK) and β-actin (1:2000; Sigma, St Louis, MO, USA). The membranes were then treated with horseradish peroxidase-conjugated secondary antibodies, and the proteins were detected using the ECL Prime Western Blotting Detection System (GE Healthcare, Tokyo, Japan) and quantified using an Image Quant LAS 4000 instrument (GE Healthcare Life Sciences).

### RNA extraction and RT–qPCR

Transfected cells were incubated for 72 h. Total RNA was extracted from cells using the RNeasy Plus Mini Kit and QIAshredder (Qiagen, Hilden, Germany), and the quantity of isolated RNA was measured using an ND-1000 spectrophotometer (NanoDrop Technologies, Wilmington, DE, USA). RT–qPCR was performed using the GoTaq 1-Step RT–qPCR System (Promega, Madison, WI, USA) in a total volume of 10 µl. The program included four stages: RT at 37°C for 15 min; RT inactivation and hot-start activation at 95°C for 10 min; 45 cycles of qPCR at 95°C for 10 s, 62°C for 10 s, and 72°C for 10 s; and dissociation at 67°C–97°C. For all RT–qPCR analyses, GAPDH mRNA was used to normalize RNA inputs. The sequences of the primer pairs were as follows: RAB5 forward, 5′-TGGGATACAGCTGGTCAAGA-3′; RAB5 reverse, 5′-GGACTTGCTTGCCTCTGAAG-3′; E-cadherin forward, 5′-GAACGCATTGCCAC ATACAC-3′; E-cadherin reverse, 5′-AGCACCTTCCATGA CAGACC-3′; N-cadherin forward, 5′-CCATCACTCG GCTTAATGGT-3′; N-cadherin reverse, 5′-ACCCA CAATCCTGTCCACAT-3′, GAPDH forward, 5′-AAGGT GAAGGTCGGAGTCAAC-3′; and GAPDH reverse, 5′-CTTGATTTTGGAGGGATCTCG-3′.

### Proliferation assay

Cell proliferation analysis was performed using the Cell Counting Kit-8 (Dojindo Laboratories, Kumamoto, Japan). At 24 h after RAB5 siRNA transfection, the SUIT-2 cells were plated in 96-well plates in 100 µl of medium containing 10% FBS at approximately 2000 cells per well. Evaluations were performed at 0, 24, 48, and 72 h. For quantifying cell viability, 10 µl of cell counting solution was added to each well and incubated at 37°C for 2 h 30 min. Next, the absorbance of the well was detected at 450 nm using the xMark^TM^ Microplate Absorbance Spectrophotometer (Bio Rad, Hercules, CA, USA).

### Invasion assay

Cell invasion assays were conducted using the 24-well BD BioCoat Matrigel Invasion Chamber (Becton Dickinson, San Jose, CA, USA) to evaluate the cellular invasion potency. SUIT-2 cells (2.0 × 10^5^ cells) were seeded in the upper chamber, and the lower chamber was filled with 750 µl of RPMI 1640 medium supplemented with 10% FBS as a chemoattractant. After 48 h of incubation at 37°C and 5% CO_2_, the cells were fixed with 70% ethanol and stained with Diff-Quick (Sysmex corporation, Kobe, Japan). The cells that invaded through the 8-µm pores to the lower surface of the filter were counted using a microscope (All-in-One BZ-X710; KEYENCE Corporation, Osaka, JAPAN; 200 magnification). In total, 10 random fields were evaluated in triplicate assays.

### Wound healing assay

We examined migration using SUIT-2 cells transfected with a negative control or RAB5 siRNA. Transfected SUIT-2 cells were grown in six-well plates until confluence, and a uniform straight wound was produced in the monolayer in each well using a pipette tip. The wells were washed with PBS to remove all the cell debris, and the cells were cultured in 5% CO_2_ at 37°C. Closure or filling in of the wound was evaluated at 50 h using a bright-field microscope (Nikon TMS; Nikon, Tokyo, Japan; 400 magnification).

### Statistical analysis

Statistical analysis of the immunohistochemical staining was performed using the χ^2^ test and Mann–Whitney *U* test. Survival curves for the patients were calculated using the Kaplan–Meier method and analyzed using the log-rank test. Prognostic factors were examined by univariate analyses using a Cox proportional hazards model. All differences were statistically significant at the level of *P* < 0.05, and a tendency was indicated at the level of *P* < 0.1. All statistical analyses were performed with JMP software, version 5.01 (SAS Institute Inc., Cary, NC, USA).

## SUPPLEMENTARY MATERIALS FIGURES AND TABLES


