# Supplementary material for: Association of RAB5 overexpression in pancreatic cancer with cancer progression and poor prognosis via E-cadherin suppression
Source: Oncotarget. 2017 Jan 17;8(7):12290–300. doi: 10.18632/oncotarget.14703 (PMC5355344; doi:10.18632/oncotarget.14703)
Supplement: Supplementary file 1 [file oncotarget-08-12290-s001.pdf]

Association of RAB5 overexpression in pancreatic cancer with cancer progression and poor prognosis via E-cadherin suppression

SUPPLEMENTARY FIGURES AND TABLE

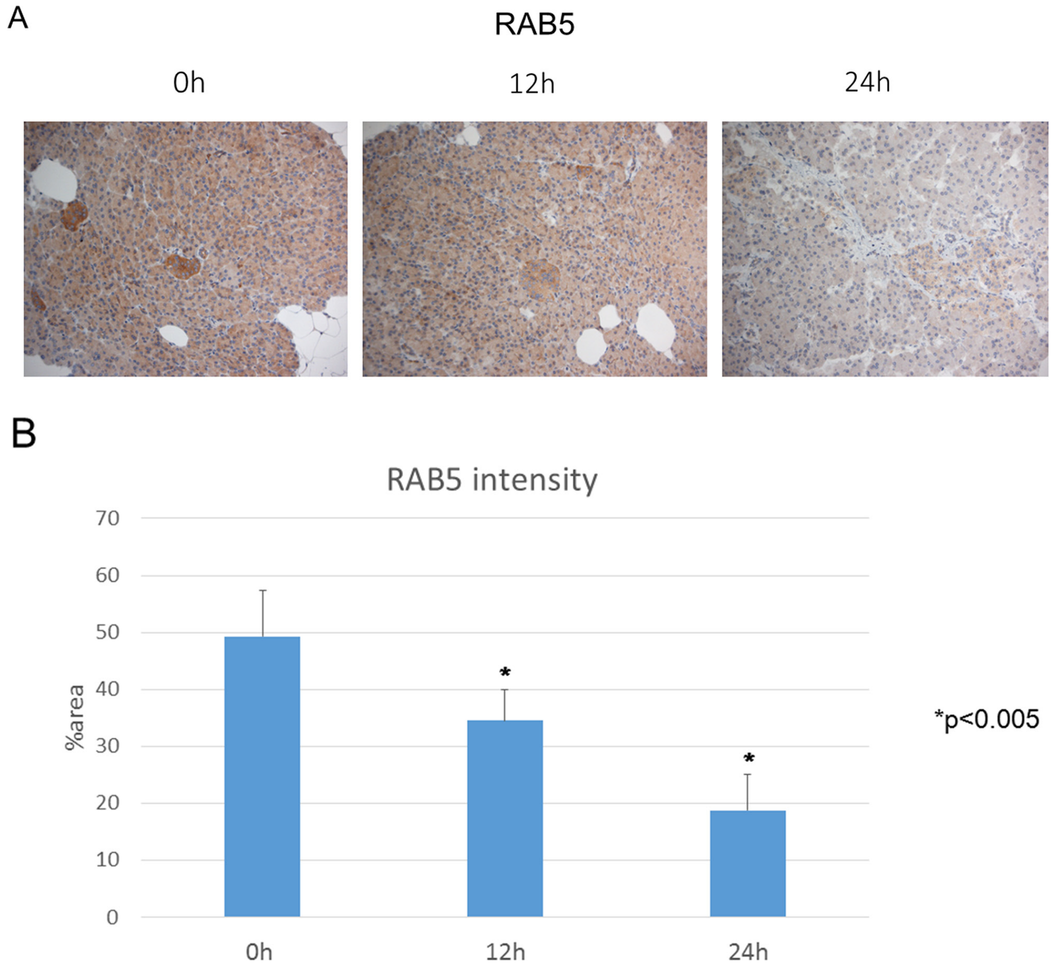

Supplementary Figure 1: Immunohistochemical staining of RAB5 in normal pancreatic tissue according to unfixed interval. A. Examples of RAB5 expression in normal pancreatic specimens: 0 h, 12 h, and 24 h elapsing between resection and formalin fixation (200×). **B.** Quantitative analysis of RAB5 expression in normal pancreatic specimens. Quantification of RAB5-positive area was performed in each normal pancreatic tissue after DAB staining. DAB-positive areas were measured using Image J 1.44 image analysis software (National Institute of Health, Bethesda, MA, USA). The amount of RAB5-positive area was calculated from 10 randomized and nonoverlapping areas (200×) (*$P < 0.05$).

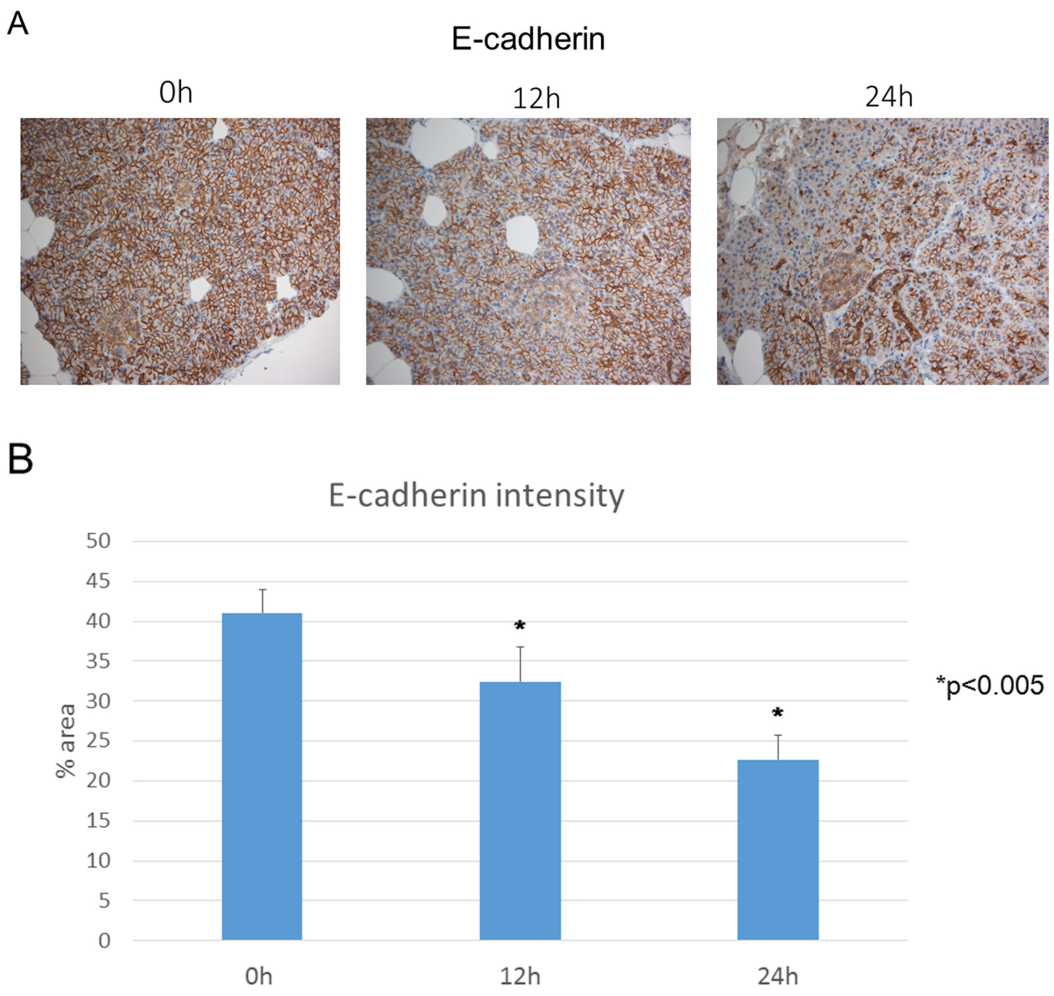

Supplementary Figure 2: Immunohistochemical staining of E-cadherin in normal pancreatic tissue according to unfixed interval. A. Examples of E-cadherin expression in normal pancreatic specimens: 0 h, 12 h, and 24 h elapsing between resection and formalin fixation (200×). **B.** Quantitative analysis of E-cadherin expression in normal pancreatic specimens. Quantification of E-cadherin-positive area was measured using Image J 1.44 image analysis software (National Institute of Health, Bethesda, MA, USA). The amount of E-cadherin-positive area was calculated from 10 randomized and non-overlapping areas (200×) (*$P < 0.05$).

Supplementary Table 1: Evaluation scores of RAB5 and E-cadherin defined by subtracting the tumor center score from the peri-frontal area score

Score	Number of patients	
	RAB5	E-cadherin
-2	0	3
-1	5	45
0	45	60
1	39	3
2	15	0
3	7	0

*Bold lines indicate that cut-off scores of the high expression group and low expression group.
